# What Is My Neuron Doing? Commentary on Huang et al. (2026)*

**DOI:** 10.1523/ENEURO.0049-26.2026

**Published:** 2026-02-24

**Authors:** Michael A. McDannald

**Affiliations:** Department of Psychology & Neuroscience, Boston College, Chestnut Hill, Massachusetts 02467

Behavioral neuroscientists are in the business of linking neuron function to behavior. Historically, single-unit recording has done much of the heavy lifting in this work. Nobel Prize-winning work on the visual system came from experiments such as Hubel and Wiesel (1962), in which 303 cortical neurons were painstakingly recorded across 40 subjects. Single-neuron firing was examined under a wide range of visual conditions. Careful evaluation of single-neuron firing, combined with anatomy and connectivity, uncovered the functional organization of the visual system. “What is my neuron doing?” is one of the most important and powerful questions in behavioral neuroscience.

Modern neuroscience has seen a torrent of new tools for monitoring neural activity. Single-unit recording can now track hundreds of neurons per session (Steinmetz et al., 2021; Song et al., 2024). Emerging voltage indicators are providing direct optical readouts of membrane potential in behaving subjects (Hao et al., 2024). Yet no technique has become more widespread than calcium imaging. Its use in behavioral neuroscience continues to accelerate, and 2025 was a banner year (Fig. 1). This commentary, as well as the article it discusses (Huang et al., 2026), focuses specifically on GCaMP-based microendoscopic imaging. Calcium imaging has major upsides, including simultaneous access to hundreds of neurons and the ability to target genetically or projection-defined neuron types. However, when data collection is complete—and 10,000+ neurons have been recorded—the age-old question remains: “What is my neuron doing?”

**Figure 1.**
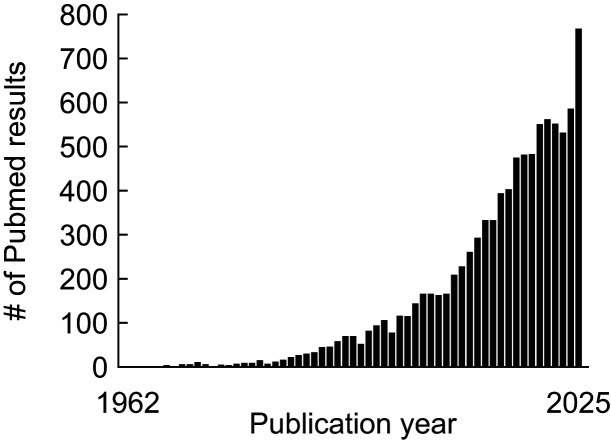
Prevalence of calcium imaging and behavior over time. A PubMed search for “calcium imaging and behavior” was performed on 2/8/2026. The numbers of results per years are reported from 1962 (the year of Hubel and Weisel foundational visual system/recording study) to 2025 (the most recent complete year).

Arguably, answering this question has never been more difficult. Microendoscopic calcium imaging does not measure firing; it measures changes in fluorescence. Fluorescence changes arise from fluctuations in intracellular calcium detected by GCaMP-class indicators. The time course of calcium entry/clearance and the kinetics of the fluorescent sensor are far slower than the millisecond dynamics of action potentials. Intense postprocessing is inherent to calcium imaging data. The initial decision to use calcium imaging sets in motion a cascade of statistical and analytical decisions. Most of these decisions are not obvious to all experimenters and are not detailed in publications—as if hidden behind a curtain.

Huang et al. pull back the curtain on these decisions. Using calcium imaging data collected from cortical neurons during elevated zero maze exploration, they systematically dissect the myriad decisions embedded in a typical analysis pipeline. They vary parameters at every stage: how neural activity is represented (continuous calcium traces, discrete events, or event-convolved signals), how behavior is quantified (head-centric vs body-centric position), how data are temporally organized (fine vs coarse binning), how sampling across states is handled (matched or unmatched), and how statistical significance is assessed (four different shuffling strategies). Across 120 decision combinations, they find that the functional label assigned to a neuron (e.g., open-arm preferring vs closed-arms preferring) shifts dramatically. As decisions accumulate, the same raw imaging data can be processed in myriad analysis combinations to support strikingly different interpretations of what individual neurons are doing. The result is eye-opening. Conclusions about neuron-specific functions depend more on analysis decisions than we typically acknowledge.

Huang et al. do not simply identify this variability; they provide a principled path forward. Drawing on model-selection logic, they evaluate each parameter combination in terms of accuracy (the ability to correctly classify neurons whose behavioral relationships are unmistakable by visual inspection) and robustness (the stability of those classifications when other parameters are perturbed). This dual-criterion framework yielded a clear, empirically grounded conclusion: Huang and colleague's most reliable setting used 2 s convolved calcium events; head-centric behavioral tracking; 50 ms binning, nonmatched state samples; and random permutation shuffling. This combination not only performed best within their selectivity-index approach; it also generalized to a regression-based framework that evaluated instantaneous calcium activity. In other words, it produced selectivity labels that were consistent even when switching to a fundamentally different analysis.

Of course, the optimal parameter configuration identified by Huang et al. is specific to their experimental conditions: GCaMP6f imaging of CaMKII-positive cortical neurons during elevated zero-maze exploration. Their parameter configuration is not intended as a universal solution for all calcium imaging studies. The value in their work lies in the framework they develop for identifying optimal parameters, not the parameters themselves. This framework provides a generalizable strategy that other laboratories can apply to their own datasets, which are certain to vary in species, cell-type investigated, calcium indicator of choice, and behavior setting.

Widespread adoption of calcium imaging has the potential to accelerate our understanding of the neural basis of behavior. At the same time, widespread adoption comes with a clear pitfall. We risk losing the ability to answer the field's most fundamental question: “What is my neuron doing?” Approaches like those of Huang et al. are imperative when using calcium imaging to answer this question.

## Synthesis

Reviewing Editor: Mihaela Iordanova, Concordia University - Loyola Campus Decisions are customarily a result of the Reviewing Editor and the peer reviewers coming together and discussing their recommendations until a consensus is reached. When revisions are invited, a fact-based synthesis statement explaining their decision and outlining what is needed to prepare a revision will be listed below. The following reviewer(s) agreed to reveal their identity: NONE.

Hi Mike,

The commentary is absolutely excellent. It is so well written, engaging and really drives the point of the paper. I just spotted on line 61 what appears to be an extra 'clear'. Perhaps you could edit that during proofs.

Mihaela
